# Over-the-Counter Oral Contraceptive Use and Initiation of Contraception

**DOI:** 10.1001/jamanetworkopen.2025.27438

**Published:** 2025-08-18

**Authors:** Maria I. Rodriguez, Haley Burns, Reed Sheridan, Alison B. Edelman

**Affiliations:** 1 Center for Reproductive Health Equity, Oregon Health & Science University, Portland

## Abstract

**Importance:**

The US Food and Drug Administration approved the first over-the-counter (OTC) oral contraceptive pill (OCP) in 2023, potentially expanding access to contraception. Understanding whether OTC availability reaches individuals with systemic barriers to care is essential to assessing its public health impact.

**Objective:**

To evaluate whether the newly available OTC progestin-only OCP is reaching individuals not previously using contraception or who rely on less-effective methods.

**Design, Setting, and Participants:**

This cross-sectional study used baseline data from a national, prospective US cohort. Participants were individuals aged 15 to 45 years obtaining OCPs at pharmacies or online between April 2024 and February 2025. Participants were eligible if they obtained an OCP (prescription or OTC) for contraceptive use and were at risk of pregnancy.

**Exposure:**

Obtaining an OTC progestin-only OCP vs a prescription OCP.

**Main Outcomes and Measures:**

Primary outcomes were (1) initiation of contraception among individuals previously not using any modern method and (2) transition from a less-effective method (eg, condoms or emergency contraception) to OCP use. Multivariable logistic regression models with robust SEs were used to determine adjusted absolute estimated probabilities and percentage point differences in outcomes.

**Results:**

The sample included 986 individuals, predominantly single (692 participants [70.2%]), and the largest proportion was aged 20 to 24 years (338 participants [34.3%]). OTC OCP users accounted for 320 (32.5%) of the participants. Individuals using the OTC option were more likely to be uninsured (101 participants [31.6%] vs 23 participants [3.5%]) and to reside in rural areas (46 participants [14.4%] vs 56 participants [8.4%]) compared with prescription users. Groups did not differ in fertility preferences. OTC use of contraception was associated with a 31.8 percentage point increase (95% CI, 24.1-39.5 percentage points) in moving from nonuse of contraception to the OTC pill, when compared with prescription OCP users. Similarly, OTC access was associated with a 41.0 percentage point increase (95% CI, 33.8-48.1 percentage points) in transitioning from use of a less-effective method of contraception to the OTC pill.

**Conclusions and Relevance:**

In this cross-sectional study of US individuals obtaining oral contraception, the OCP was accessed OTC by many who previously used no method or less-effective contraception. These findings suggest that OTC access may improve equitable access to contraception.

## Key Points

**Question:**

Is access to an over-the-counter (OTC) oral contraceptive pill (OCP) associated with improved contraceptive initiation among US individuals with structural barriers to care?

**Findings:**

In this cross-sectional study of 986 oral contraceptive users, individuals relying on OTC access were more likely than prescription users to be uninsured, adolescent, and living in rural areas. OTC access resulted in a 31.8 percentage point increase in individuals shifting from no contraceptive method to an effective method, a significant difference.

**Meaning:**

These findings suggest that OTC OCP may improve access to effective contraception in the US.

## Teaser

This cross-sectional study evaluates whether the newly available over-the-counter progestin-only oral contraceptive pill in the US is associated with increased contraceptive use among individuals with systemic barriers.

## Introduction

In 2023, the US Food and Drug Administration approved the first over-the-counter (OTC) birth control pill for use in the US. Oral contraceptive pills (OCP), both progestin-only and combined, are safe, effective, and easy to use—all qualities that have made them good candidates for consideration of OTC status.^1^ More importantly, OTC availability is one way to improve contraceptive access by removing barriers to initiation, continuation, and adherence or correct use, all of which may aid in decreasing the rate of unintended pregnancy.^2^

In the US, OCPs are the most common form of reversible contraception used.^3^ Almost 80% of US reproductive aged female individuals who ever had sexual intercourse with a male partner have used a birth control pill in their lifetime.^4^ Globally, OTC OCPs are currently available in more than 100 countries, some with access for over 50 years.^5^ Data from global use and recent actual use clinical studies in the US show that consumers can easily self-screen and use progestin-only contraceptive pills.^6,7^

The OTC OCP became widely available to buy in stores and online in April of 2024.^1^ This availability became notably important as possible threats to Title X and Medicare by the Trump administration have the potential to limit the accessibility of prescribed contraceptive methods.^8^ The uninsured and those living in areas with limited access to health centers may be disproportionately affected, further increasing their barriers to contraceptive information and services.

We sought to evaluate whether the OTC OCP is associated with improved access for individuals who desire to avoid pregnancy with structural barriers to care, such as adolescents, those living in rural areas, and uninsured individuals. The objective of our study was to evaluate whether the new availability of an OTC OCP was reaching people who had either not previously been contracepting or were using a less-effective method of contraception.

## Methods

This cross-sectional study used data from the baseline wave of a prospective cohort study of individuals obtaining OCPs in pharmacies and online across the US. The full study is powered to detect a 9.5% difference in contraceptive continuation rates at 12 months. All analyses and reporting of results were conducted in accordance with Strengthening the Reporting of Observational Studies in Epidemiology (STROBE) reporting guidelines.^9^ The Oregon Health & Science University institutional review board approved the study protocol, and all study participants provided informed consent.

### Study Population

We recruited people aged 15 to 45 years who presented to pharmacies or online retailers to obtain the OCP. We partnered with a national pharmacy chain to recruit people from 63 pharmacies across 31 states in 2 ways. To recruit people using OCP provided by prescription, individuals filling a contraceptive prescription were handed study materials by a pharmacy team member. We placed labels with QR codes linking to our online survey on packs of the OTC OCP in these stores. The OTC OCP was available on the shelves of these drug stores, not behind the counter at the pharmacy. We also worked with Opill.com to recruit people purchasing the OTC OCP online. After purchasing the OTC OCP, individuals were directed to a study information page. Finally, we used Build Clinical to generate participant referrals. Build Clinical is a clinical trial recruiting system that helps investigators recruit participants for clinical trials more efficiently. They used study-specific digital advertisements displayed on search engines, health websites, and social media platforms to identify potential participants who were then screened by study team members.

Participants needed to be biologically capable of pregnancy (eg, no history of menopause, hysterectomy, or salpingectomy). Individuals receiving any type of OCP by prescription, or by purchasing the progestin-only pill (Opill; Perrigo) OTC were eligible for enrollment. We recruited in English and Spanish. We excluded individuals who were using the pill for noncontraceptive indications or were not at risk of pregnancy (not sexually active, have permanent contraception, or infertility).

### Variables

We collected demographic, health history, pregnancy intention, and preference information from each participant. All demographic data were self-reported. Demographic data included age, race and ethnicity, zip code of residence, level of education, insurance, relationship status, whether they were born in the US, and employment status. Age was reported in years. Self-reported race and ethnicity were captured using the standard National Institutes of Health categories and using a format that allowed individuals to select as many racial and/or ethnic identities as applied to them. Data on race and ethnicity were collected because of reported differences in contraceptive access and use. We used the participant’s zip code to determine urban or rural residence, using Rural Urban Commuting Area codes.^10^ We captured the region of the US where a participant resided (Northeast, Midwest, South, West).^11^ Education was classified as a categorical variable, with options ranging from no schooling to completion of a doctoral degree. Respondents were asked whether they had health insurance, and the type of plan was recorded (commercial, Medicaid, Medicare, other plan, or uninsured). We classified relationship status as single, never married; married or partnered; widowed; divorced; or separated.

We asked respondents how many pill packs were obtained and what method of contraception they had been using immediately before the visit. We captured whether individuals had a history of pregnancy and what the outcome was. Data on the frequency of vaginal intercourse with a partner with sperm were recorded. We specifically sought to elicit information about pregnancy intention, as individuals planning pregnancy within the year may use contraception differently. Respondents were asked whether they planned a pregnancy within the next year (yes, no, or unsure). Those answering no or unsure were asked how important it was to them to avoid a pregnancy (extremely important, important, or open to pregnancy). We also assessed reasons for using the OTC OCP. For individuals using the OTC OCP, we asked for the top reason they selected it.

### Outcomes

We examined how the availability of an OTC OCP changed contraceptive use in our cohort. We specifically sought to understand whether the OTC OCP was reaching people at highest risk of unintended pregnancy—that is, sexually active individuals who were not using any modern method or were using a less-effective method of contraception. We looked at changes in contraceptive use by examining the method a person reported using immediately before their pharmacy visit and the method they used at the visit end. We compared people using the OTC OCP with those receiving an OCP by prescription. Our study outcomes were (1) moving from nonuse of a method to use of the OCP and (2) moving from use of a less-effective method to use of the OCP. We classified contraceptive use or nonuse in the following way: (1) OCP, contraceptive patch, contraceptive ring, injectable, implant, or intrauterine device as moderate or highly effective methods; (2) condoms and emergency contraception as less-effective methods; and (3) nonuse of a method (no method or not using a modern method, rhythm method or natural family planning, or lactational amenorrhea) before the pharmacy visit.

### Statistical Analysis

We first examined demographic and clinical characteristics between people using prescription and OTC OCP. Given geographic variations in contraceptive access, we mapped the ratio of study participants using the OTC to prescription OCP by state to visualize the residence of study participants.^12^ We created quartiles from the ratio of OTC to prescription OCP users for each state, with quartile 1 representing the least OTC OCP compared with prescription OCP use, and quartile 4 representing the most OTC OCP compared with prescription OCP use. States were categorized as not applicable if there were no OTC or prescription OCP users from that state. We then visualized these quartiles overlaid onto a US map.

We estimated multivariable logistic regression models with robust SEs to estimate the probability of our binary outcomes of interest (moving from nonuse of contraception and moving from use of a less-effective method of contraception). To aid in interpretation of the results, we present both absolute and relative effect measures of adjusted odds ratios, adjusted estimated probabilities, and percentage point differences in probabilities, with 95% CIs for each. Adjusted estimated probabilities were based on the model and were averaged over the covariates using a sample-based weighted average. All models were adjusted for rurality, whether it was extremely important to avoid a pregnancy, and age.

All statistical analyses were performed using Stata statistical software version 18 (StataCorp), and mapping was done using R statistical software version 4.2.3 (R Project for Statistical Computing). A 2-tailed *P* value was used for all analyses with an α level of .05 to indicate statistical significance.

## Results

**Figure 1.**
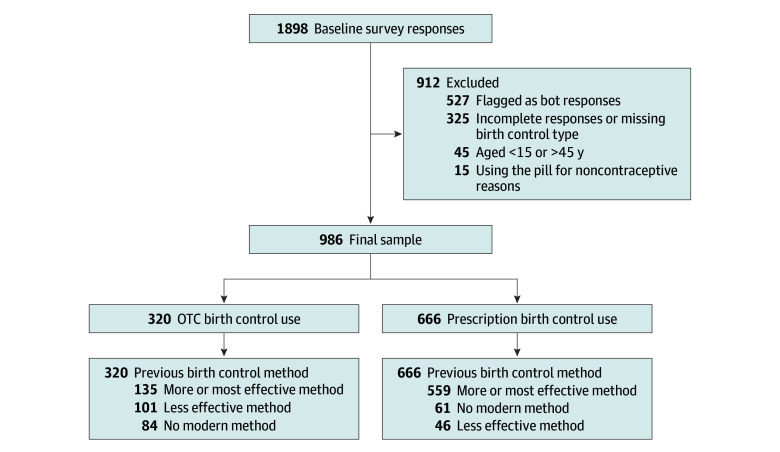
Study Enrollment Flowchart OTC indicates over the counter.

Overall, our sample included 986 individuals (Figure 1), predominantly single (692 participants [70.2%]), and the largest proportion was aged 20 to 24 years (338 participants [34.3%]). With regard to race and ethnicity, 24 individuals (2.4%) were American Indian or Alaska Native, 131 individuals (13.3%) were Asian, 97 individuals (9.8%) were Black, 116 individuals (11.8%) were Hispanic or Latina, 12 individuals (1.2%) were Native Hawaiian or Pacific Islander, 721 individuals (73.1%) were White, and 1 individual (0.1%) was of unknown race and ethnicity (Table 1). OTC OCP users accounted for 32.5% (320 participants) of our study sample (Table 1). In this convenience sample, a higher proportion of adolescents (aged 15-20 years) were using the OTC OCP compared with a prescription method (45 participants [14.1%] vs 43 participants [6.5%]) (Table 1). More people who were uninsured were using the OTC OCP than were using a prescription method (101 participants [31.6%] vs 23 participants [3.5%]). More people living in rural areas were using the OTC OCP than the prescription OCP (46 participants [14.4%] vs 56 participants [8.4%]). The OTC OCP group also included more individuals who identified as Black or Latina, with lower educational attainment, and from the Southern US.

**Table 1.** Demographics of a National Sample of Oral Contraceptive Users by Method of Contraceptive Access

Characteristic	Participants, No. (%)		
	Over the counter (n = 320 [32.5%])	Prescription (n = 666 [67.5%])	Total (N = 986 [100.0%])
Age group, y			
<20	45 (14.1)	43 (6.5)	88 (8.9)
20-24	79 (24.7)	259 (38.9)	338 (34.3)
25-29	67 (20.9)	169 (25.4)	236 (23.9)
30-34	62 (19.4)	84 (12.6)	146 (14.8)
35-39	38 (11.9)	73 (11.0)	111 (11.3)
40-45	29 (9.1)	38 (5.7)	67 (6.8)
Race and ethnicity^a^			
American Indian or Alaska Native	9 (2.8)	15 (2.3)	24 (2.4)
Asian	17 (5.3)	114 (17.1)	131 (13.3)
Black	50 (15.6)	47 (7.1)	97 (9.8)
Hispanic or Latina	53 (16.6)	63 (9.5)	116 (11.8)
Native Hawaiian or Pacific Islander	5 (1.6)	7 (1.1)	12 (1.2)
White	217 (67.8)	504 (75.7)	721 (73.1)
Unknown	1 (0.3)	0	1 (0.1)
Sexuality			
Straight	231 (72.2)	480 (72.1)	711 (72.1)
Lesbian or gay	4 (1.2)	11 (1.7)	15 (1.5)
Bisexual	65 (20.3)	137 (20.6)	202 (20.5)
Another term or unknown	17 (5.3)	34 (5.1)	51 (5.2)
Unspecified	3 (0.9)	4 (0.6)	7 (0.7)
Education			
Less than high school	14 (4.4)	4 (0.6)	18 (1.8)
High school diploma or General Educational Development	86 (26.9)	81 (12.2)	167 (16.9)
Some college or associate’s degree	107 (33.4)	146 (21.9)	253 (25.7)
Bachelor’s degree	75 (23.4)	278 (41.7)	353 (35.8)
Graduate or professional degree	38 (11.9)	157 (23.6)	195 (19.8)
Born outside the US	30 (9.4)	43 (6.5)	73 (7.4)
Rural location	46 (14.4)	56 (8.4)	102 (10.3)
Region of the US			
Northeast	55 (17.2)	103 (15.5)	158 (16.0)
Mideast	62 (19.4)	131 (19.7)	193 (19.6)
South	147 (45.9)	167 (25.1)	314 (31.8)
West	56 (17.5)	265 (39.8)	321 (32.6)
Marital status			
Single, never married	206 (64.4)	486 (73.0)	692 (70.2)
Married or domestic partnership	82 (25.6)	159 (23.9)	241 (24.4)
Widowed, divorced, or separated	32 (10.0)	21 (3.2)	53 (5.4)
Occupational status			
Employed or self-employed	204 (63.7)	399 (59.9)	603 (61.2)
Out of work, looking for work	34 (10.6)	27 (4.1)	61 (6.2)
Out of work, not currently looking for work	5 (1.6)	3 (0.5)	8 (0.8)
Stay-at-home parent or caretaker	21 (6.6)	23 (3.5)	44 (4.5)
Student	52 (16.2)	212 (31.8)	264 (26.8)
On disability and unable to work	4 (1.2)	2 (0.3)	6 (0.6)
Insurance			
Commercial	133 (41.6)	523 (78.5)	656 (66.5)
Medicaid	47 (14.7)	69 (10.4)	116 (11.8)
Medicare	14 (4.4)	13 (2.0)	27 (2.7)
Other plan	7 (2.2)	18 (2.7)	25 (2.5)
Uninsured	101 (31.6)	23 (3.5)	124 (12.6)
Unknown	18 (5.6)	20 (3.0)	38 (3.9)

^a^ This was a select-all-that-apply question, so percentages may not add up to 100%.

**Figure 2.**
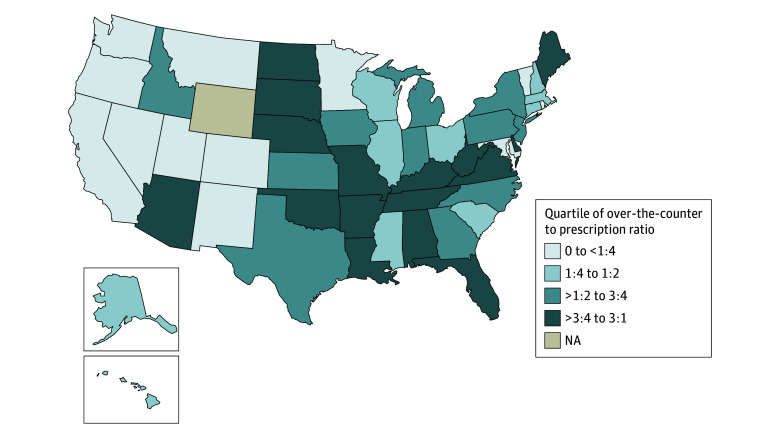
Distribution of Study Population of Over-the-Counter to Prescription Pill Use Ratio by State (N = 986) NA indicates not applicable.

Our map comparing OTC with prescription OCP use revealed geographic trends in our study population. The majority of states in the West (8 of 13 states [61.5%]) were in quartile 1 of OTC to prescription ratio (less than 1 OTC user to every 4 prescription users), whereas less than 10% of states in the West (1 of 13 states [7.7%]) were in quartile 4 (more than 3 OTC users for every 4 prescription users) (Figure 2). Conversely, less than 10% of states in the South (1 of 17 states [5.9%]) were in quartile 1 of the OTC to prescription ratio, with the majority of states (10 of 17 states [58.8%]) in quartile 4. The states of Arkansas, Delaware, Florida, Kentucky, Louisiana, and Oklahoma in the South had an OTC to prescription ratio greater than 1, reflecting more OTC users compared with prescription users. Most Mideast and Northeast states were in quartiles 2 or 3 of OTC to prescription use.

We similarly observed differences in reproductive characteristics between people using the OTC OCP and those who obtained OCPs by prescription. More users relying on the OTC OCP had a history of pregnancy (104 participants [32.5%] vs 124 participants [18.6%]; χ^2^ = 23.43; *P* ≤ .001) and of giving birth (91 participants [28.4%] vs 98 participants [14.7%]; χ^2^ = 29.47; *P* ≤ .001) (Table 2). OTC OCP users reported more frequent rates of vaginal intercourse than prescription users. OTC OCP users were significantly less likely to have received contraceptive counseling within the last 12 months (124 participants [38.8%] vs 402 participants [60.4%]), and to be using any modern method of contraception at baseline (84 participants [26.2%] vs 61 participants [9.2%] using no modern method; χ^2^ = 223.51; *P* ≤ .001). Of all OCP users, 42.2% (135 of 320 users) were already using a more or most effective method of contraception immediately before their pharmacy visit, compared with 83.9% of prescription contraceptive users (559 of 666 users) (Figure 1). We did not find any significant difference between groups in planning a pregnancy within the next 12 months, or the importance of avoiding pregnancy. The most common reasons reported for using the OTC OCP included not requiring an appointment (76 of 320 participants [23.8%]), not having a regular physician (59 of 320 participants [18.4%]), and efficiency (47 of 320 participants [14.7%]; data not shown).

**Table 2.** Reproductive Measures by a National Sample of Oral Contraceptive Users by Method of Contraceptive Access

Characteristic	Participants, No. (%)		
	Over the counter (n = 320 [32.5%])	Prescription (n = 666 [67.5%])	Total (N = 986 [100.0%])
Obstetric characteristics			
History of pregnancy (yes)^a^	104 (32.5)	124 (18.6)	228 (23.1)
History of birth (yes)^a^	91 (28.4)	98 (14.7)	189 (19.2)
History of abortion (yes)	23 (7.2)	39 (5.9)	62 (6.3)
History of ectopic pregnancy (yes)	1 (0.3)	2 (0.3)	3 (0.3)
History of miscarriage (yes)	30 (9.4)	45 (6.8)	75 (7.6)
Planning to become pregnant in next 12 mo (yes)	6 (1.9)	15 (2.3)	21 (2.1)
Openness to pregnancy in the next 12 mo			
Extremely important to avoid	252 (78.8)	544 (81.7)	796 (80.7)
Important to avoid	46 (14.4)	64 (9.6)	110 (11.2)
Open to pregnancy	21 (6.6)	58 (8.7)	79 (8.0)
Unspecified	1 (0.3)	0	1 (0.1)
Vaginal intercourse frequency^a^			
Daily	22 (6.9)	10 (1.5)	32 (3.2)
2-4 Times per week	102 (31.9)	181 (27.2)	283 (28.7)
Weekly	101 (31.6)	176 (26.4)	277 (28.1)
Monthly	35 (10.9)	129 (19.4)	164 (16.6)
Rarely or never	60 (18.8)	170 (25.5)	230 (23.3)
Had contraceptive counseling within the last 12 mo^a^	124 (38.8)	402 (60.4)	526 (53.3)
What contraceptive method were you using before today?^a^			
No method	84 (26.2)	61 (9.2)	145 (14.7)
Condoms	87 (27.2)	45 (6.8)	132 (13.4)
Emergency contraception	14 (4.4)	1 (0.2)	15 (1.5)
Contraceptive patch, pill, or ring	92 (28.7)	503 (75.5)	595 (60.3)
Injectable	5 (1.6)	8 (1.2)	13 (1.3)
Implant	2 (0.6)	6 (0.9)	8 (0.8)
Intrauterine device	8 (2.5)	9 (1.4)	17 (1.7)
Vasectomy	5 (1.6)	2 (0.3)	7 (0.7)
Abstinence	23 (7.2)	31 (4.7)	54 (5.5)

^a^ Pearson χ^2^ *P* ≤ .001.

On the basis of our multivariable models, OTC OCP users had estimated probabilities of nearly 50% of moving from use of both no contraception (42.4%; 95% CI, 35.2%-49.6%) and a less-effective method of contraception (49.2%; 95% CI, 42.4%-55.7%) (Table 3). Compared with the reference group of prescription OCP users, OTC users were estimated to be on average 31.8 percentage points more likely to move from nonuse of contraception (95% CI, 24.1-39.5 percentage points), and 41.0 percentage points more likely to move from a less-effective contraceptive method (95% CI, 33.8-48.1 percentage points).

**Table 3.** Association of Over-the-Counter Pill Use With New Use of Contraception

Variable	Moving from nonuse of contraception (n = 778)^a^^,^^b^			Moving from less-effective contraception (n = 780)^a^^,^^b^		
	aOR (95% CI)	Estimated probability, % (95% CI)	Difference, percentage point (95% CI)^c^	aOR (95% CI)	Estimated probability, % (95% CI)	Difference, percentage point (95% CI)^c^
Prescription	1 [Reference]	10.6 (8.1-13.1)	NA	1 [Reference]	8.1 (5.9-10.4)	NA
Over the counter	6.52 (4.34-9.80)	42.4 (35.2-49.6)	31.8 (24.1-39.5)	12.37 (7.89-19.37)	49.2 (42.4-55.7)	41.0 (33.8-48.1)

Abbreviations: aOR, adjusted odds ratio; NA, not applicable.

^a^ Models were adjusted for rurality, extremely important to avoid pregnancy, and age.

^b^ No method indicates no modern method and includes no method, withdrawal, natural family planning or rhythm method, and lactational amenorrhea; less-effective method included condoms and emergency contraception; respondents who indicated prior method of abstinence and vasectomy excluded.

^c^ Percentage difference refers to the percentage point difference in the adjusted estimated probability between those using over-the-counter birth control and the reference of prescription birth control.

## Discussion

In this cross-sectional study of people with a biologic capacity for pregnancy in the US obtaining OCPs either via traditional prescription pathways or directly OTC, the availability of an OTC OCP was associated with improved contraceptive uptake among individuals at elevated risk of unintended pregnancy.^13^ Notably, the OTC OCP reached a significant proportion of individuals who were not previously using any contraceptive method or who relied on less-effective options, such as condoms or emergency contraception. These findings suggest that the OTC OCP may serve as a critical access point for contraceptive initiation and method switching, particularly for populations facing structural barriers to care.

Importantly, OTC OCP users in our sample were more likely to be uninsured and reside in rural areas, underscoring the promise of OTC availability in bridging gaps where health care access is limited.^14^ These findings are particularly salient in the context of ongoing threats to reproductive health care access under proposed federal and state-level policy changes.^8,15,16,17^ Pregnancy is dangerous in the US; there are high rates of maternal mortality and sharp disparities by racial and socioeconomic lines.^18,19^ With the overturn of federal protections for abortion rights in the US, with the *Dobbs v Jackson* decision, and decreased access to safe abortion, maternal morbidity and mortality are likely to increase.^20,21,22^ Women from minoritized racial and ethnic groups, adolescents, the uninsured, and Medicaid recipients all experience higher rates of maternal morbidity and mortality.^23,24^ We found that the OTC OCP is being used at higher rates by these populations, suggesting it may help reduce preventable maternal morbidity by reducing unintended pregnancies. Our findings are similar to data from Mexico, where OTC OCPs are also a critical source of access for adolescents and the uninsured.^25^

Identifying opportunities to facilitate contraceptive choice is critical; the Coalition to Expand Contraceptive Access and others have identified that the current contraceptive workforce cannot meet current or future demand.^26^ Emerging evidence demonstrates that *Dobbs v Jackson* has affected both abortion and contraceptive access, through clinic closures and changes in the contraceptive workforce.^15,27,28,29^ Compounding this, it is widely anticipated that the Trump administration will shortly reinstate the policies implemented in its first term, including sharp reductions in funding to Title X clinics, a major backbone of publicly funded contraception in the US.^30^ Our study provides a first look at who is using OTC OCPs, as the method has been available in the US for only approximately a year. We found that more than one-half of all those purchasing the OTC OCP for pregnancy prevention were not using a method or were relying on a less-effective method for contraception. Given that this was a group where the clear majority expressed it was extremely important to avoid pregnancy in the next year, and were having vaginal intercourse regularly, our finding of a 31.8 percentage point increase in moving from no method to the OCP suggests that the OTC OCP is helping people meet their reproductive goals.

To ensure that the OTC OCP is truly available and accessible for all who need it, work to implement insurance reimbursement of OTC contraceptive methods is needed.^31,32^ Current pricing is approximately $50 for a 3-month supply.^33^ Cost has historically been a major barrier to accessing one’s preferred method of contraception.^34^ Although currently federal law requires most private health plans and Medicaid to cover without cost sharing the full range of Food and Drug Administration–approved contraceptives, less is known on how coverage occurs for methods that are not prescribed. Six states have passed laws requiring public insurance plans to cover OTC contraception without a prescription, and another 7 use state-only Medicaid funds to provide this coverage for Medicaid recipients. Preliminary findings suggest that although there is broad stakeholder support, key barriers to insurance coverage for OTC methods exist. These barriers include limited outreach to contraceptive users and staff, inconsistent reimbursement mechanisms, and federal policy barriers that further constrain equitable implementation, particularly within Medicaid and self-funded private insurance plans.

### Limitations

Our study should be interpreted with the following limitations in mind. First, although we adjusted for key covariates, unmeasured confounding may persist, particularly regarding fertility preferences or sexual behavior. Importantly, we found no difference between groups regarding the importance of avoiding a pregnancy in the next year, although OTC users of contraception were slightly more sexually active. Second, self-selection into OTC vs prescription methods may reflect underlying differences in health care engagement or contraceptive preferences that we were unable to fully capture. In addition, this analysis reflects early uptake following a change in method availability; longer-term data will be needed to assess continuation, adherence, and impact on unintended pregnancy rates.

Despite these limitations, our findings support the conclusion that OTC availability of OCPs is reaching populations with significant unmet need and enabling the choice of transitioning to more effective contraceptive use. As policymakers and public health professionals consider the role of OTC contraception in the broader reproductive health landscape, these data underscore the value of expanding and sustaining access to this modality. Future research should examine long-term outcomes, including contraceptive continuation and unintended pregnancy rates, and evaluate insurance reimbursement for the OTC OCP.

## Conclusions

In this national cohort study, the OTC availability of a progestin-only OCP was associated with increased uptake among individuals at high risk of unintended pregnancy, including those who were previously nonusers or using less-effective methods. These findings suggest that removing prescription requirements for OCPs can expand contraceptive access, particularly for underserved populations, such as the uninsured and those in rural areas. As reproductive health care access faces growing threats, OTC contraception offers a promising strategy to support reproductive autonomy and reduce disparities in contraceptive use. Improving community awareness of this option and insurance reimbursement policies will be essential to ensure equitable access and sustained impact.

## Notes

Supplement.
Data Sharing Statement
