# Supplementary material for: Over-the-Counter Oral Contraceptive Use and Initiation of Contraception
Source: JAMA Netw Open. 2025 Aug 18;8(8):e2527438. doi: 10.1001/jamanetworkopen.2025.27438 (PMC12362221; doi:10.1001/jamanetworkopen.2025.27438)
Supplement: Supplement. — Data Sharing Statement [file jamanetwopen-e2527438-s001.pdf]

Data Sharing Statement

Rodriguez. Over-the-Counter Oral Contraceptive Use and Initiation of Contraception. *JAMA Netw Open*. Published August 18, 2025. doi:10.1001/jamanetworkopen.2025.27438

Data
Data available: No
